# Ferroptosis in central nervous system injuries: molecular mechanisms, diagnostic approaches, and therapeutic strategies

**DOI:** 10.3389/fncel.2025.1593963

**Published:** 2025-07-22

**Authors:** Qiuhu Huang, Haowen Zhang, Shenbo Chen, Yao Wang, Jian Zhou

**Affiliations:** Department of Neurosurgery, The First Affiliated Hospital of Hainan Medical University, Haikou, China

**Keywords:** neurological injuries, ferroptosis, iron, lipid peroxidation, central nervous system

## Abstract

Ferroptosis, an iron-dependent form of cell death, has emerged as a critical factor in the pathogenesis of central nervous system (CNS) injuries, including neurodegenerative diseases, stroke, and traumatic brain injury. This review highlights disrupted iron metabolism, glutathione depletion, and antioxidant system impairment as core mechanisms, alongside polyunsaturated fatty acid oxidation contributing to neuronal damage. Diagnostic advancements, such as MRI-based iron quantification and lipid ROS detection, offer clinical potential but require validation. Therapeutic strategies, including iron chelators, antioxidants, and lipid metabolism modulators, demonstrate efficacy in preclinical models by attenuating ferroptosis. Translational challenges persist due to incomplete mechanistic insights, tissue-specific iron dynamics, and delivery limitations. The dual role of iron in CNS physiology and pathology underscores the need for interdisciplinary research to refine diagnostics and therapies. Emphasizing ferroptosis as a therapeutic target, this work advocates for a deeper exploration of immune interactions and combinatorial approaches to improve outcomes in CNS injuries.

## 1 Introduction

Central nervous system (CNS) injuries, including neurodegenerative diseases and stroke, pose a significant global health burden as the leading cause of disability and the second most common cause of death globally (Chin and Vora, 2014). The pathological basis of these conditions involves aberrant neuronal death, exacerbated by neurons’ limited capacity for regeneration through cellular division. Neuronal death occurs through multiple pathways including apoptosis, necrosis, pyroptosis, parthanatos, and the recently discovered ferroptosis (Fricker et al., 2018). While these pathways share some common features, ferroptosis displays distinct morphological and biochemical characteristics that differentiate it from other forms of cell death.

Ferroptosis, first described in 2012, is an iron-dependent form of regulated cell death characterized by: (1) accumulation of lipid peroxides and reactive oxygen species (ROS), (2) distinct mitochondrial morphology including shrinkage and cristae reduction, and (3) dependence on iron metabolism (Dixon et al., 2012; Yao et al., 2021). Ferroptosis is mostly caused by glutathione peroxidase 4 (GPX4) failure and consequent lipid peroxidation (LP), as opposed to apoptosis, which requires caspase activation, or necrosis, which results in cell death. Ferroptosis, a novel form of cell death in CNS injuries, has garnered increasing attention due to its underlying mechanism and role (Dixon et al., 2012; Dixon, 2017). The pathological basis of CNS injuries lies in aberrant neuronal death, which is compounded by neurons’ inability to divide, thus preventing the replacement of damaged neurons through standard cellular renewal mechanisms. Notably, the brain, the most oxygen-consuming organ in the human body, has relatively weak antioxidant defense mechanisms and is rich in polyunsaturated fatty acids (PUFAs) (Kagan et al., 2017; Wu et al., 2019). This makes the brain especially vulnerable to ferroptosis and more susceptible to its damaging effects. Increasing evidence indicates a strong association between ferroptosis and CNS injuries, particularly in crucial CNS regions such as the cerebral cortex, hippocampus, striatum, and spinal cord (Dixon et al., 2012; Skouta et al., 2014; Chen et al., 2015; Martinez et al., 2019). Therefore, studying ferroptosis is crucial for advancing the treatment and rehabilitation of CNS injuries.

Iron, a vital trace element, is essential for the proper functioning of the CNS (Thirupathi and Chang, 2019). However, excessive iron accumulation can lead to neuronal death through ferroptosis and exacerbated inflammation (Xiong et al., 2019). Abnormal iron accumulation is closely associated with the progression of various CNS injuries, including Parkinson’s disease (PD) (Xiong et al., 2019) and Alzheimer’s disease (AD) (Peng et al., 2021), stroke, and traumatic brain injury (TBI). Consequently, understanding the mechanism and role of ferroptosis in CNS injuries is vital to developing new diagnostic methods and therapeutic strategies, potentially offering significant clinical value for improving the prognosis and outcomes.

While the role of ferroptosis in CNS injuries has been initially recognized, the underlying mechanisms remain incompletely understood (Goldsteins et al., 2022; David et al., 2023). Moreover, many studies are limited to animal models and *in vitro* experiments. To comprehensively analyze existing evidence, we systematically searched PubMed and Web of Science using the following keyword combinations: ferroptosis AND (central nervous system injury)/ferroptosis AND (central nervous system damage)/ferroptosis AND (central nervous system disease)/ferroptosis AND (neurodegenerative diseases)/ferroptosis AND [(Parkinson’s disease) OR (Alzheimer’s disease) OR (Huntington’s disease) OR (multiple sclerosis)]/ferroptosis AND [(subarachnoid hemorrhage) OR (SAH)]/ferroptosis AND [(traumatic brain injury) OR TBI OR (head injury)]/ferroptosis AND [stroke OR (cerebral hemorrhage)]/ferroptosis AND (spinal cord injury). However, the papers that were not reviews or research articles were not included. This article summarizes the mechanisms, diagnostic approaches, and therapeutic strategies for ferroptosis in CNS injuries. We conduct a comprehensive review of recent studies on ferroptosis and CNS injuries, aiming to enhance our understanding of the underlying mechanisms and to refine therapeutic strategies for treating CNS injuries.

## 2 Ferroptosis

### 2.1 Definition and characteristics of ferroptosis

In Dolma et al. (2003) discovered that erastin could effectively induce tumor cell death in RAS-mutant tumors. They found that this process did not involve traditional cell death pathways (Dolma et al., 2003). Iron chelators could suppress this death process, accompanied by increased intracellular lipid ROS levels (Yagoda et al., 2007; Yang and Stockwell, 2008). The term “ferroptosis” was introduced by Dixon et al. (2012) to describe a distinctive form of cell death that is iron-dependent and non-apoptotic. This process is characterized by the buildup of intracellular lipid ROS (Dixon et al., 2012). These findings suggest that ferroptosis may represent a novel mechanism of cell death. Cell death is a complex and diverse process, and ferroptosis, as a unique mode of cell death, differs significantly from necrosis, apoptosis, and autophagy in terms of morphology, biochemical characteristics, and gene expression (Stockwell et al., 2017; Wu et al., 2018). Ferroptosis is defined by the preservation of functional cytoplasmic membranes, increased mitochondrial membrane density, decreased or absent cristae, increased release of oxidized PUFAs, elevated cytoplasmic levels of lipid ROS, and the process can be inhibited by iron chelators (Yang and Stockwell, 2008; Dixon et al., 2012).

### 2.2 Molecular mechanisms of ferroptosis

#### 2.2.1 Iron metabolism

Iron is an essential physiological element, and its distribution and levels can significantly impact physiological processes (Yan and Zhang, 2019). Iron overload plays a critical factor in ferroptosis. Iron (Fe^2+^) generates large amounts of hydroxyl radicals through Fenton and iron-catalyzed Haber-Weiss reactions, catalyzing the generation of ROS, which in turn leads to the development of LP (MacKenzie et al., 2008; Bogdan et al., 2016). Fe^2+^ is produced through intestinal absorption or erythrocyte degradation and then oxidizes to iron (Fe^3+^). Fe^3+^ binds to transferrin and enters cells via transferrin receptor (TFR) mediated endocytosis (Frazer and Anderson, 2014). Within endosomes, Fe^3+^ is reduced back to Fe^2+^ primarily by six-transmembrane epithelial antigen of prostate 3 (STEAP3) *in vivo* (Ohgami et al., 2006; Bogdan et al., 2016), with significant contribution from superoxide anion (O_2_^•–^) generated through NADPH oxidase activity. This reduction is critical for Fenton reactions: Fe^2+^ reacts with H_2_O_2_ to generate hydroxyl radicals (•OH) via the iron-catalyzed Haber-Weiss reaction (Liochev and Fridovich, 1999). Iron can be stored in the labile iron pool or transported via divalent metal transporter 1 (DMT1) or Zinc-Iron regulatory protein family 8/14. Excessive Fe^2+^ is oxidized to Fe^3+^ by ferroportin (FPN) to maintain intracellular iron homeostasis (Bogdan et al., 2016). Silencing TFR can inhibit erastin-induced ferroptosis (Gao et al., 2015), while heme oxygenase-1 (HO-1) supplementation accelerates erastin-induced ferroptosis (Kwon et al., 2015). Heat shock protein beta-1 reduced intracellular iron by suppressing telomeric repeat factor 1, further inhibiting ferroptosis (Sun et al., 2015). Ferritin, a 24-subunit nanocage complex comprising ferritin light chain (FTL) and ferritin heavy chain 1 (FTH) polypeptides, functions as the primary intracellular iron storage protein to inhibit ferroptosis at the cellular level through two key mechanisms: (1) iron sequestration: the ferroxidase activity of FTH causes up to 4,500 iron atoms to mineralize within its cavity and oxidize Fe^2+^ to Fe^3+^. This lowers the pool of labile iron that is accessible for Fenton reactions (Arosio et al., 2017). (2) antioxidant protection: Ferritin inhibits iron-catalyzed lipid peroxidation by separating redox-active iron. FTH1 overexpression gives resistance to erastin-induced ferroptosis, whereas genetic ablation of FTH1 increases cellular vulnerability (Alvarez et al., 2017). Systemically, serum ferritin represents the body’s iron storage, but because it is largely an iron buffer and cannot release iron, it does not directly control ferroptosis (Wang et al., 2010). The ferroptosis-inhibitory function is thus confined to the intracellular compartment. Fe^2+^ is transported to Fe^3+^ for storage in ferritin through FPN or poly(rC)-binding protein 1/2, depending on the formation of heteropolymers by FTH and FTL.

Iron homeostasis begins with dietary absorption in the duodenum, where Fe^3+^ is reduced to Fe^2+^ by duodenal cytochrome B and transported via DMT1 into enterocytes. Systemic iron is oxidized to Fe^3+^ by hephaestin, bound to transferrin, and circulated in plasma. Excess iron is stored in hepatocytes or macrophages via ferritin, while systemic efflux is regulated by ferroportin (FPN) and hepcidin (Frazer and Anderson, 2014). Endosomal Fe^3+^ is reduced to Fe^2+^ by STEAP3 and exported to the cytosol via DMT1. Excess Fe^2+^ is exported by FPN or oxidized by ceruloplasmin. Disruption of these regulatory mechanisms elevates labile iron pools, potentiating Fenton reactions and ferroptosis.

#### 2.2.2 Amino acid metabolism

Mounting studies indicate that cystine uptake via System Xc^–^ is crucial for maintaining glutathione (GSH) levels and inhibiting ferroptosis (Aoyama and Nakaki, 2015). Inhibiting GSH synthesis can induce ferroptosis (Yang et al., 2014). System Xc^–^ (a special cystine/glutamate transporter) exchanges intracellular glutamate for extracellular cystine in a 1:1 ratio (Dixon et al., 2012), driven by the high intracellular glutamate concentration rather than adenosine triphosphate (Bridges et al., 2012; Friedmann Angeli et al., 2014). The System Xc^–^ transport may be inhibited by elevated extracellular glutamate levels. Following cellular uptake, cystine undergoes reduction to cysteine, primarily mediated by thioredoxin-related protein 14 (TRP14) through the dithiol-disulfide exchange (Martí-Andrés et al., 2024). This cysteine then reacts with glutamate, forming γ-glutamylcysteine, a process catalyzed by glutamylcysteine ligase. Finally, glycine combines with γ-glutamylcysteine to produce GSH, facilitated by the enzyme GSH synthetase (Philpott and Ryu, 2014). The function of the anti-LP enzyme GPX4 can be directly inhibited, through covalent modification by compounds like RSL3 (RAS-selective lethal 3), or indirectly through GSH depletion, leading to the generation of excessive lipid peroxides, causing ferroptosis (Cui et al., 2022; Weaver and Skouta, 2022). This process is the critical factor in ferroptosis. GPX4 consumes GSH to reduce lipid hydroperoxides (L-OOH) to lipid alcohols. At the same time, GSH is oxidized to GSH disulfide, which is then decreased back to GSH by nicotinamide adenine dinucleotide phosphate (NADPH)-dependent on GSH reductase to enter the next cycle.

Additionally, L-OOH is oxidized by Fe^2+^ to generate tremendously reactive lipid alkoxyl radicals (LO•), which destroy PUFAs through a chain reaction, leading to membrane injury and cell death (Gaschler and Stockwell, 2017). GSH can also act as a binding ligand for Fe^2+^ in the unstable iron pool, preventing the formation of hydroxyl radicals from its reaction with H_2_O_2_ (Philpott and Ryu, 2014). GPX4 utilizes GSH to reduce lipid peroxides of PUFAs to lipids, maintaining the balance between lipid peroxides and lipids.

#### 2.2.3 Lipid metabolism

Fatty acids are essential molecular constituents of the brain, comprising over 50% of its dry weight, with PUFAs representing approximately 40% of the total FAs (Lauritzen et al., 2001; Yao et al., 2021). PUFAs are integral components of neuronal membrane phospholipids, playing a crucial role in sustaining essential membrane functions, including signal transduction, regulation of ion channels, and the activity of membrane-associated proteins (Wainwright, 2002; Haag, 2003). By modulating synaptic activity, neurotransmitter levels, and receptor distribution and activity, PUFAs directly impact cognitive function, learning, and memory in the brain (Chalon, 2006; Weiser et al., 2016).

**FIGURE 1:**
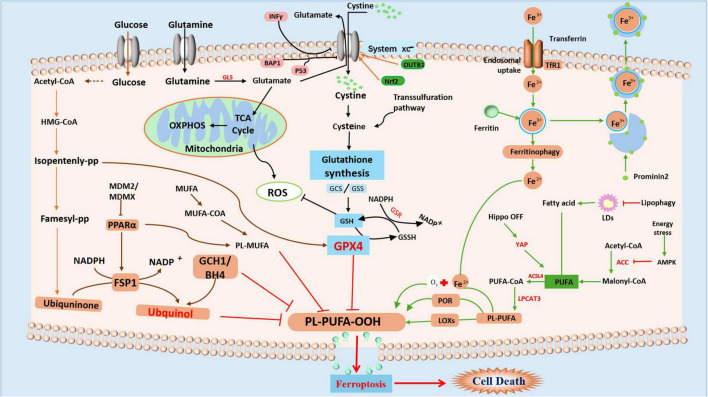
Summary of the mechanism of ferroptosis Ferroptosis is mainly caused by the GSH-dependent enzyme GPX4, which inhibits the production of Fe^2+^dependent lipid ROS by converting cytotoxic lipid hydroperoxides (L-OOH) to non-toxic alcohols (L-OH). Controlled by the cystine/glutamate antiporter (System Xc^–^), GSH functions as a cofactor for GPX4. Ferroptosis is brought on by depleting GSH, inactivating GPX4, and raising lipid peroxides when System Xc^–^, GSH production, or GPX4 itself are inhibited. Iron overload results from a disturbance of iron homeostasis, which includes ferritinophagy-mediated breakdown, absorption, export, and storage. Through the Fenton reaction, excess iron catalyzes the production of harmful lipid ROS. Furthermore, PUFAs are changed into peroxidation-susceptible PUFA phospholipids by enzymes (such as COXs and LOXs).

Omega-3 PUFAs possess distinct anti-inflammatory and metabolic characteristics, suggesting their significant potential for treating PD and AD (Cunnane et al., 2009; Weiser et al., 2016), and multiple sclerosis (MS) (Siegert et al., 2017). Docosahexaenoic acid (DHA), an Omega-3 PUFA, has been proven to effectively inhibit abnormal neuronal firing in epilepsy by regulating voltage-gated potassium channels (Börjesson and Elinder, 2011). PUFAs are essential for neurological maturation during early development, especially in infancy; a deficiency of DHA precursors has been associated with occipital cortex dysfunction and peripheral neuropathy (Uauy et al., 2000). However, the high reactivity of PUFA makes them vulnerable to free radical attack and LP, resulting in a high risk of OS on brain tissue. Particularly vulnerable are PUFAs containing allyl carbon, such as arachidonic acid and DHA, which are easily oxidized to harmful LP products (Uauy et al., 2000). Under pathological conditions, phosphatidylethanolamine, derivatives of PUFAs, may undergo oxidation mediated by lipoxygenases, resulting in the formation of lipid peroxides, which can trigger ferroptosis (Uauy et al., 2000). The disruption of cellular membrane integrity, particularly in neuronal cell membranes and organelles such as mitochondria and the endoplasmic reticulum (Gaschler and Stockwell, 2017; Kagan et al., 2017; Zou et al., 2020), adversely impacts neural functions by altering membrane potential and disrupting ion homeostasis. This ultimately adds to neuronal dysfunction and death by causing abnormal synaptic activity and reduced neurotransmitter release. In particular, Omega-3 PUFAs such as DHA, by altering cell membrane fluidity, may directly influence cellular sensitivity to oxidative stress and thus the process of ferroptosis. The cellular metabolic mechanisms of ferroptosis are illustrated in Figure 1.

#### 2.2.4 Other metabolic pathways

Mitochondria play an essential role in the advancement and development of ferroptosis (Gao et al., 2019). The mevalonate pathway affects the production of CoQ10, an endogenous antioxidant involved in regulating iron metabolism. Levels of NADPH and selenium also influence the development of ferroptosis. NAD PH has a reducing effect, while selenium promotes the biosynthesis of GPX4, which enhances resistance to iron-induced damage. Glutamine metabolic pathways are crucial for the formation of intracellular iron ions and ROS, thereby regulating iron metabolism. Glutaminase transforms glutamine, a key substrate for the tricarboxylic acid cycle (TCA), into α-ketoglutarate (α-KG). In addition to improving mitochondrial respiration and electron transport chain activity, α-KG replenishes TCA cycle intermediates, which raises the production of ROS (Martínez-Reyes and Chandel, 2020; Zhang et al., 2021). In addition, α-KG can be utilized via malic enzyme to generate NADPH, a process that supports glutathione regeneration (Cluntun et al., 2017). α-KG activates prolyl hydroxylases (PHDs) that stabilize hypoxia-inducible factor 1α (HIF-1α) (Martínez-Reyes and Chandel, 2020). HIF-1α transcriptionally upregulates TfR1, leading to increased iron uptake (Yang et al., 2018). Additionally, HIF-1α increases ceruloplasmin, which promotes the oxidation of Fe^2+^ to Fe^3+^, facilitating the efflux of iron from cells (Yang et al., 2025). Glutamine provides carbon skeletons for the manufacture of glutathione through the enzyme glutamate-cysteine ligase and selenocysteine biosynthesis, which is essential for GPX4 action (Ingold et al., 2018). Thus, glutamine metabolism serves as a metabolic rheostat for ferroptosis sensitivity by coordinately regulating iron homeostasis and oxidative stress.

### 2.3 Ferroptosis and other cell death pathways

Ferroptosis differs from other cell death modalities in both molecular mechanisms and morphological characteristics, since it is a unique iron-dependent type of controlled cell death triggered by LP and GPX4 inactivation (Table 1).

**TABLE 1:** Key differences of cell death pathways.

Feature	Ferroptosis	Apoptosis	Necroptosis	Autophagy
Molecular triggers	Iron overload	DNA damage	TNF-a signaling	Nutrient deprivation
	GSH depletion	Extrinsic signals	Caspase-8 inhibition	ER/mitochondrial stress
	GPX4 inhibition	Intrinsic stress	Viral infection	mTOR inhibition
	Lipid peroxidation			
Key regulatory proteins	GPX4	Caspases (3, 8, 9)	RIPK1/RIPK3	ATG proteins
	System Xc^–^	Bcl-2 family	MLKL	Beclin-1
	ACSL4	p53	CYLD	LC3-II
	NRF2			
Morphological features	Mitochondrial shrinkage	Cell shrinkage	Organelle swelling	Autophagosome formation
	Loss of cristae	Nuclear fragmentation	Plasma membrane rupture	Lysosomal degradation
	Preserved nuclear integrity	Apoptotic bodies	Inflammatory release	Cytoplasmic vacuoles
Biochemical hallmarks	Iron-dependent ROS accumulation	Caspase activation	Phosphorylation of MLKL	LC3 lipidation
	Lipid ROS accumulation	DNA fragmentation	Membrane permeabilization	Degradation of cytoplasmic components
Immune response	Pro-inflammatory (DAMP release)	Anti-inflammatory (non-immunogenic)	Pro-inflammatory (cytokine release)	Context-dependent (pro-survival or pro-death)
Key inhibitors	Ferrostatin-1	Z-VAD-FMK (pan-caspase inhibitor)	Necrostatin-1 (RIPK1 inhibitor)	3-methyladenine (PI3K inhibitor)
	Liproxstatin-1	Bcl-2 overexpression	GSK′872 (RIPK3 inhibitor)	Chloroquine (lysosomal inhibitor)
	Iron chelators (DFO)			

#### 2.3.1 Ferroptosis and necroptosis

The process of necroptosis is governed by the activation of receptor-interacting protein kinases (RIPK) 1/3, along with mixed lineage kinase domain-like protein (MLKL) (Conrad et al., 2016). Müller et al. (2017) found in a mouse model of renal ischemia-reperfusion injury that the absence of ACSL4, a factor associated with ferroptosis, rendered cells more vulnerable to necroptosis over time. In contrast, cells lacking in MLKL protein were more prone to ferroptosis. Additionally, aberrant RIPK3, p-MLKL, FTL, and LP expression were observed in mouse hippocampal tissues in a chronic mild unpredictable stress model (Cao et al., 2021). However, further research is essential to clarify the regulatory interactions between necroptosis and ferroptosis.

#### 2.3.2 Ferroptosis and autophagy

Autophagy is a cellular process characterized by the formation of autophagosomes, which are double-membrane structures that encapsulate cytoplasmic constituents and organelles for degradation. These autophagosomes subsequently fuse with lysosomes to form autolysosomes, where the sequestered contents are degraded to meet cellular metabolic needs and facilitate organelle turnover (Luo and Tao, 2020). Recent studies have demonstrated that heat shock protein 90, a critical molecular chaperone, regulates Lamp-2a expression in the autophagic pathway and regulates ferroptosis (Zhang C. et al., 2024). Nuclear receptor coactivator 4 (NCOA 4) serves as a selective cargo receptor that targets ferritin to autophagosomes (Le et al., 2024). This NCOA4-mediated degradation releases iron stores, increasing labile iron pools (Hoelzgen et al., 2024; Zhu et al., 2024). Silencing NCOA4 reduces iron-mediated neuronal death in TBI models (Li et al., 2021; Bengson et al., 2023). Another study found that autophagy promoted the degradation of neuronal ferritin in a mouse model of SAH, leading to elevated free iron levels and ultimately promoting ferritin deposition (Liang et al., 2022). These results deepen our insight into the interplay between autophagy and ferroptosis. However, additional investigation is necessary to clarify the regulatory mechanisms that connect these two processes.

#### 2.3.3 Ferroptosis and apoptosis

Apoptosis is a common mechanism of programmed cell death, which can be initiated by external or internal pathways. It has been found that the ferroptosis inducer, erastin, irreversibly activates CHOP/PUMA within the p53 signaling pathway, thereby enhancing the sensitivity of tumor cells to apoptosis inducers and facilitating apoptosis (Hong et al., 2017). These findings provide important clues for exploring the interaction between ferroptosis and apoptosis. This process is characterized by several distinct features: nuclear condensation, cell swelling, and the emergence of lipid membrane vesicles on the plasma membrane, ultimately resulting in cell lysis, all occurring without fragmentation of nuclear DNA (Hu et al., 2020).

#### 2.3.4 Ferroptosis and pyroptosis

Pyroptosis is a form of inflammatory cell death that is typically triggered by pathogen-associated molecular patterns or damage-associated molecular patterns. It is characterized by cell swelling, plasma membrane rupture, and the release of pro-inflammatory cytokines such as interleukin-1β and interleukin-18 (Bergsbaken et al., 2009; Broz, 2025). Pyroptosis is caused by the activation of inflammasomes, which then trigger caspase-1, cleaving gasdermin D and creating membrane holes. These pores allow the discharge of intracellular chemicals, like ROS, which can worsen lipid peroxidation and oxidative stress (Bergsbaken et al., 2009). Recent studies have indicated that there may be crosstalk between pyroptosis and ferroptosis (Cao et al., 2022; Eskander et al., 2025). Mitochondrial dysfunction links ferroptotic lipid peroxidation to pyroptotic NLRP3 activation (Chen et al., 2023; Lyamzaev et al., 2023; Tian et al., 2023). Ferroptosis may be facilitated by a pro-oxidant environment created by the generation of ROS and the loss of cellular membrane integrity during pyroptosis. Ferroptosis may also be further promoted by the release of iron from injured cells during pyroptosis, which may worsen iron excess in the surrounding tissue. However, further research is needed to determine the precise molecular processes and signaling pathways that underlie the connection between these two types of cell death.

## 3 Role of ferroptosis in CNS injuries

**FIGURE 2:**
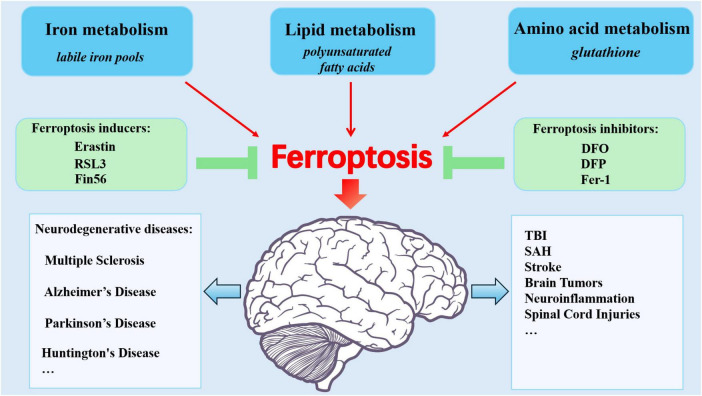
Ferroptosis: Metabolic Pathways, Modulators, and CNS injury diseases. Ferroptosis stems from dysregulated iron metabolism, lipid metabolism, and amino acid metabolism. The inhibitors and inducers are involved in ferroptosis. Brain iron overload contributes to CNS injuries including stroke, TBI, SAH, tumors, neuroinflammation, spinal cord injuries, and neurodegenerative diseases.

Ferroptosis is closely related to CNS injuries, with current research focusing mainly on neurodegenerative diseases, stroke, TBI, and similar conditions (Figure 2).

### 3.1 Parkinson’s disease

Parkinson’s disease (PD) is mainly defined by the gradual degeneration of dopamine-producing neurons located in the iron-rich substantia nigra. Mitochondrial and lysosomal dysfunction, dysregulated iron metabolism, and accumulation of ROS are considered to be pivotal elements in the pathogenesis of PD, ultimately leading to neuronal degeneration and death (Matak et al., 2016; Burbulla et al., 2017). Epidemiological studies show that high dietary iron intake may increase the risk of PD. Ferroptosis, a novel form of cell death regulated by protein kinase C (PKC), plays a critical role in PD pathogenesis. PKC activation increases the catalytic activity of 12/15-lipoxygenase toward PUFAs in neuronal membranes by phosphorylating it. This encourages ferroptotic death and lipid peroxidation (Choudhary et al., 2022; Pratt, 2023). Overexpression of the PKCδ isoform increases the susceptibility of substantia nigra neurons, but PKC inhibitors such as chelerythrine decrease the α-synuclein aggregation in dopaminergic cells caused by rotenone (Kabiraj et al., 2015). Recent studies have highlighted the significant role of glial cells, including microglia and astrocytes, in the progression of PD (Wang et al., 2023; Brash-Arias et al., 2024). Studies have identified excessive iron accumulation, alongside activated microglia and immunoreactive astrocytes, in the brains of individuals with PD (Lee and Lee, 2019; Foley et al., 2022; López-Aguirre et al., 2025). This glia can induce neuronal death by triggering OS and releasing proinflammatory factors (Braak et al., 2007). Susceptibility-weighted imaging (SWI) exploits magnetic susceptibility differences between tissues to quantify non-heme iron. SWI has been used to assess iron deposition in the basal ganglia of PD patients, with findings indicating a progressive increase as the disease advances (Mohammadi and Ghaderi, 2024; He et al., 2025; Lancione et al., 2025). Ferrostatin-1 (Fer-1) analogs and PKC inhibitors have proven effective in mitigating neuronal injury caused by ferroptosis. Deferoxamine (DFO) alleviates PD symptoms, especially in patients with reduced plasma ceruloplasmin (Kosyakovsky et al., 2021). Ceruloplasmin, a ferroxidase enzyme, is a significant blood protein that carries copper and contributes to iron metabolism by promoting the oxidation of Fe^2+^ to Fe^3+^, which is necessary for iron storage and transportation (Roeser et al., 1970; Liu et al., 2022). DFO improves motor symptoms in PD patients with low ceruloplasmin levels by lowering oxidative stress and iron overload (Grolez et al., 2015; Lin et al., 2022). DFO effectively reduces iron content in the dentate nucleus regions without adversely affecting cognition or mood (Martin-Bastida et al., 2017). However, the overall efficacy of DFO in PD remains uncertain, highlighting the need for more extensive clinical trials to explore the potential benefits of iron chelation therapy. Fer-1 has also been found to inhibit ROS/RNS, attenuating rotenone-induced α-synuclein aggregation in dopaminergic SH-SY5Y cells (Kabiraj et al., 2015), thereby reversing MPTP-triggered behavioral deficits in a mouse model of PD and protecting the neurons (Do Van et al., 2016). Aggregation of alpha-synuclein in human-induced pluripotent stem cell-derived neurons promotes calcium influx and LP, thereby triggering ferroptosis (Angelova et al., 2020). Notably, three distinct inhibitors of ferroptosis, including PUFAs, DFO, and Fer-1, effectively impede this process, thus creating new therapeutic possibilities for PD treatment.

### 3.2 Alzheimer’s disease

Alzheimer’s disease (AD) is marked by the gradual deterioration of neurons within the CNS. Accumulating evidence indicates that iron imbalance triggers OS and the overproduction of ROS, subsequently leading to cell death—a process indicative of the involvement of ferroptosis in AD progression (Raven et al., 2013). The reduction of GPX4 severely impairs neuronal defenses against OS and increases the risk of ferroptosis. In GPX4 brain-inducible knockout mouse models, vitamin E deficiency accelerated hippocampal neuronal degeneration and behavioral decline (Wortmann et al., 2013). At the same time, inhibition of ferroptosis with Liproxstatin-1 (Lip-1) slowed this process, highlighting the effect of ferroptosis in AD and suggesting novel therapeutic avenues (Hambright et al., 2017). Molecules with anti-ferroptosis properties significantly protect the CNS, providing a new perspective for AD administration. Many studies have focused on α-lipoic acid (LA), a small molecule with antioxidant and iron-chelating properties. LA inhibits Tau-mediated imbalances in iron metabolism, LP, and inflammatory responses in P301S tau transgenic mice (Zhang Y. H. et al., 2018). It also activates the mitogen-activated protein kinases signaling pathway and promotes GPX4 expression, enhancing resistance to ferroptosis and decreasing the accumulation of neurofibrillary tangles. Chalcone derivatives, particularly compounds 14a-c, have demonstrated protective solid effects against Aβ1-42-induced neurotoxicity in SH-SY5Y cell models by inhibiting LP and preventing ferroptosis (Cong et al., 2019). Additionally, GSH deficiency may exacerbate OS, which in turn affects AD development. Studies have found that iron accumulation can exacerbate GSH loss, leading to increased ROS production (Núñez et al., 2004; Belaidi and Bush, 2016; Li et al., 2020). MRI testing has revealed raised iron levels in the hippocampus of AD patients (van Bergen et al., 2016). Another study has demonstrated that astrocytes and neurons mediate the interaction between ferroptosis and AD progression (Fan et al., 2024; Li et al., 2024). Hepcidin expression in astrocytes regulates iron intake, which helps mitigate neuronal loss in the cortex and hippocampus of amyloid precursor protein/presenilin-1 (APP/PS1) mice (Xu et al., 2020; Li et al., 2024). Activation of microglia and iron accumulation in APP/PS1 mice has been associated with neuroinflammation and Aβ aggregation (McIntosh et al., 2019). Thus, astrocyte-regulated iron efflux impairment, microglial iron buildup, and iron-mediated GSH depletion create a self-reinforcing cycle that propels ferroptosis in AD. This triad may be the focus of early therapies that delay neurodegeneration.

### 3.3 Huntington’s disease

Huntington’s disease (HD) is a progressive neurodegenerative disorder inherited in an autosomal dominant manner. Research indicates that superfluous iron accumulation in the brain’s basal ganglia may accelerate disease progression (Sánchez-Castañeda et al., 2013; Domínguez et al., 2016; Niu et al., 2018; Abdelfattah et al., 2020). Iron accumulation, LP, and GSH dysregulation are directly linked to ferroptosis. A study demonstrates that iron induces microglial activation in HD mice, suggesting a synergistic interaction between iron and HD in promoting this activation (Donley et al., 2021). Research has shown that HD patients’ striatal synaptosomes exhibit higher levels of 4-hydroxynonenal (4-HNE), a hallmark of LP (Lee et al., 2011; Lee et al., 2025). The striatum’s GPX4 activity is decreased in the R6/2 mouse model of HD (Chen et al., 2015). The loss of GPX4, an essential enzyme that shields cells from LP, would make neurons more vulnerable to ferroptosis (Bellinger et al., 2011; Hambright et al., 2017). Research indicates that mitochondrial GPX4 depletion increases ROS, further contributing to cellular damage and ferroptosis (Agrawal et al., 2018). Mounting studies have revealed that cell injury in HD primarily involves the loss of astrocytes, oligodendrocytes, and neurons, potentially affecting the cerebral cortex and striatum (Yu et al., 2003; Tydlacka et al., 2008; Crotti et al., 2014). The main therapeutic agents targeting HD-associated ferroptosis include DFO and Fer-1. DFO can alleviate excessive iron accumulation in the brain’s basal ganglia, while Fer-1, a ferroptosis inhibitor, prevents oxidative lipid injury and cell death in HD models (Skouta et al., 2014). These findings offer a promising new therapeutic strategy for HD.

### 3.4 Multiple sclerosis

Multiple sclerosis (MS) is a chronic inflammatory condition of CNS marked by demyelination, neuronal injury, and associated neurological dysfunction. Recent studies have highlighted the critical role of iron metabolism imbalance in the pathogenesis of MS. Ferroptosis may be a major pathogenic process in MS, as evidenced by studies showing raised iron levels and increased LP in lesion regions and cerebrospinal fluid (CSF) of MS patients, associated with impaired iron homeostasis (Zivadinov et al., 2018; Van San et al., 2023; Zhang X. et al., 2024). In mouse models of MS, decreased expression of GPX4 and SLC7A11, along with elevated LP, has been observed in gray matter and spinal cord tissues (Hu et al., 2019; Moezzi et al., 2022). These models exhibit abnormal mitochondrial morphology in neurons, consistent with features of ferroptosis. Inhibition of ferroptosis or downregulation of ACSL4 expression significantly improves clinical symptoms, reduces neuronal death, and alleviates neuroinflammation (Van San et al., 2023). Further research indicates that ferroptosis can foster T-cell activation and the development of autoimmune responses (Luoqian et al., 2022). These findings suggest that developing targeted therapies to inhibit T-cell activation mediated by ferroptosis could assist in suppressing CNS inflammation and treat MS, offering new hope for patients with this debilitating condition. Furthermore, brain organoids and three-dimensional culture models are employed for high-throughput drug screening because they can replicate the networks involved in iron metabolism in the human brain (Aldewachi et al., 2021).

### 3.5 Stroke

#### 3.5.1 Ischemic stroke

Mounting studies have revealed that ischemic stroke increases iron deposition, mitochondrial dysfunction, LP, and iron overload. Ischemia-reperfusion injury suppresses the expression of tau protein, triggers iron accumulation in neurons, and promotes ferroptosis (Tuo et al., 2017). Inhibitors of ferroptosis have the potential to improve outcomes in patients with ischemic stroke. N-acetylcysteine (NAC) protects neurons from iron-induced ferroptosis by reducing free radical production, thereby reducing neuronal mortality (Kamel et al., 2024). NAC treatment decreased the initiation of cascades of neuronal death, including lipid peroxidation, after experimental cerebral ischemia (Hong et al., 2020). A clinical trial study suggests that NAC promotes early neurological recovery in patients with ischemic stroke (Komakula et al., 2024). Carvacrol has been found to protect Gerbil hippocampal neurons from ischemia-reperfusion injury by impeding ferroptosis by increased GPX4 expression (Guan et al., 2019).

#### 3.5.2 Hemorrhagic stroke

Research has investigated that ferroptosis occurs in experimental cerebral hemorrhage (CH) models and brain tissues of patients affected by CH (Li et al., 2017; Zille et al., 2017; Chen et al., 2019). In a CH rat model, GPX4 levels gradually decreased, reaching a low point at 24 h after CH (Zhang Z. et al., 2018). After CH, free iron is released from the hemoglobin degradation products, leading to excessive iron accumulation in brain tissue. This generates high ROS, LP, and OS levels, ultimately resulting in cell death. The iron chelator DFO has been shown to decrease ROS production, mitigate hemoglobin-induced neurotoxicity, reduce brain edema, and enhance functional outcomes in both *in vivo* and *in vitro* models of CH (Regan and Panter, 1996; Nakamura et al., 2004; Okauchi et al., 2009). Additionally, Fer-1 significantly reduces secondary brain injury following CH. Potential therapeutic targets for ferroptosis after CH include selenium supplementation, iron chelators, lipoxygenase inhibitors, and DMT1 inhibitors. These studies provide valuable insight into the mechanisms underlying ferroptosis in strokes and identify potential therapeutic targets.

### 3.6 Traumatic brain injury

Traumatic Brain Injury (TBI) is followed by a cascade of secondary injuries, including the release of excitatory neurotransmitters and Fe^2+^, OS, LP, and the accumulation of ROS, all of which induce ferroptosis (Anthonymuthu et al., 2018). Iron levels in the brain significantly increased after TBI, likely due to hemorrhage-induced iron deposition in the brain parenchyma. Chronic brain injury may occur in a region of iron deposition and is associated with tissue loss. Studies have indicated that iron and ROS accumulation, increased transferrin levels, decreased GPX activity, and mitochondrial dysfunction can induce ferroptosis after TBI. Fer-1 reduces tissue damage and improves outcomes in a TBI mouse model (Xie et al., 2019). Controlled cortical impact leads to the accumulation of oxidized PE, upregulated expression of 15-lipoxygenase and ACSL4, and reduced GSH (Kenny et al., 2019). TBI research in animals mostly uses the controlled cortical impact (CCI) approach. But when it comes to mimicking secondary damage pathways and reproducing the pathological variety of genuine TBI, this model shows serious shortcomings. Accordingly, future research should concentrate on creating clinically appropriate multimodal models in order to more accurately replicate the course of disease.

Additionally, miR-212-5p regulates prostaglandin-endoperoxide synthase-2 to reduce ferroptosis-induced neuronal death (Xiao et al., 2019). In TBI mice, alterations in iron regulatory proteins (GPX4, SLC7A11, ACSL4) are evident in the brain cortex. TBI activates ferroptosis, inflammation, and immune responses, mainly through microglia and macrophages, influencing TBI outcomes and offering therapeutic targets (Li and Jia, 2023). Cells undergoing ferroptosis release pro-inflammatory damage-associated molecular patterns which activate the innate immune response. This activation disrupts the equilibrium between pro-inflammatory and anti-inflammatory processes, particularly within brain tissues (Proneth and Conrad, 2019). This disruption may exacerbate the initial injury and contribute to the progression of neurological damage. Understanding ferroptosis’s role in neuronal death post-TBI offers new avenues for therapy, highlighting the importance of researching optimal timing for anti-ferroptosis treatments and drug development.

### 3.7 Spinal cord injuries

Spinal cord injuries (SCI), known for causing severe disability and mortality, present significant medical challenges worldwide. The pathogenesis of SCI is intricate and not yet fully understood, with current treatment options being limited. Recent research suggests that activated microglia in SCI can release substantial amounts of nitric oxide, which in turn decreases ferritin levels while upregulating the expression of TFR, DMT1, and iron regulatory protein 1 in motor neurons (Feng et al., 2021). Research has revealed that iron chelators and specific inhibitors may have therapeutic potential for SCI. DFO upregulates GPX4, xCT, and GSH, curbs gliocyte overgrowth, enhances long-term motor function, and mitigates iron overload (Yao et al., 2019). SRS16-86, a new ferroptosis inhibitor, significantly elevates GPX4, GSH, and xCT levels, decreases LP, restrains glial cell activation, and reduces neuronal injury in SCI models (Zhang et al., 2019). Anthocyanin and grape seed-derived proanthocyanidins improve motor function in SCI mice by decreasing iron and TBARS levels, downregulating ALOX15 and ACSL4 while upregulating HO-1, GSH, and GPX4 (Zhou et al., 2020). Fer-1 reverses mitochondrial irregularities and inflammation after SCI by inhibiting glial cell activity, reducing iron accumulation, and downregulating the expression of ferroptosis-related genes. Zinc ions prevent ferroptosis after SCI by enhancing Nrf2/HO-1 activity and reducing ferroptosis products such as GSH, GPX4, and ROS (Ge et al., 2021). Lipoxin A4, an anti-inflammatory lipid mediator, promotes neuroprotection and recovery after SCI by influencing the Akt/Nrf2/HO-1 pathway (Lu et al., 2018; Wei et al., 2021). Edaravone is a free radical scavenger that enhances GPX4 and xCT while reducing ACSL4 and ROS, suggesting it may inhibit ferroptosis in the acute phase SCI. These compounds intervene in ferroptosis through multiple mechanisms, opening new therapeutic avenues for SCI. Ongoing studies aim to validate their clinical safety and efficacy, striving to enhance treatment options for patients with SCI.

### 3.8 Shared molecular drivers of ferroptosis in CNS injuries

Ferroptosis is a major pathogenic process in CNS injuries, such as stroke, TBI, and neurodegenerative disorders. Although there are triggers unique to each disease, the fundamental molecular processes causing ferroptosis show notable commonalities, providing a single target for treatment. We summarize these common mechanisms and how disease-specific factors affect them below.

(1) Core pathways

Excessive iron accumulation in CNS injuries drives divergent mechanisms: nigral iron excess in PD promotes LP via Fenton reactions (Ayton and Lei, 2014; You et al., 2015), while AD demonstrates co-localized hippocampal iron deposition with tau/Aβ pathology (Van San et al., 2023; Zhou et al., 2024), and stroke or TBI liberates free iron to amplify oxidative stress (Toro-Urrego et al., 2019; Liu D. et al., 2024). These processes synergize with GPX4 suppression, a critical antioxidant enzyme whose downregulation exacerbates neuronal damage by impairing lipid peroxide neutralization in AD, stroke, and TBI models (Yoo et al., 2010; Hambright et al., 2017; Weaver and Skouta, 2022). Furthermore, ACSL4-mediated esterification of PUFAs into peroxidation-vulnerable membrane domains establishes a self-reinforcing pathway, with elevated ACSL4 expression in stroke, AD, and PD intensifying lipid oxidative damage (Zheng et al., 2024), collectively forming an iron-dependent oxidative injury axis.

(2) Disease-specific modulation

Iron-bound α-synuclein oligomers in PD produce redox-active substances that hasten dopaminergic degradation and LP (Calabresi et al., 2023). While hyperphosphorylated tau interferes with mitochondrial iron export, increasing neuronal iron retention and lipid ROS through GPX4 depletion, Aβ plaques in AD trap iron to produce oxidative hotspots (Liu D. et al., 2024). Microglial cytokine-driven inhibition of System Xc^–^ and GSH depletion, in conjunction with hemorrhagic heme iron excess and blood-brain barrier (BBB) rupture, prime neurons for ferroptosis in stroke/TBI (Tang et al., 2020; Guo et al., 2023; Wei, 2024).

## 4 Diagnostic approaches for ferroptosis in CNS injuries

Ferroptosis is characterized by iron buildup, which can be measured in a number of ways. Ferroptosis diagnosis necessitates a multimodal evaluation of antioxidant failure, lipid peroxidation, and iron dysregulation. Neuroimaging, biofluid analysis, and histopathology methods can all be used to accomplish this thorough assessment.

### 4.1 Neuroimaging biomarkers

MRI has become a useful technique for evaluating brain iron buildup. The substantia nigra of PD patients and the hippocampus of AD patients can both have iron accumulation that SWI can identify (Raven et al., 2013; Xu et al., 2020). The more accurate measurement of brain iron concentration is provided by quantitative susceptibility mapping (QSM), which has shown notable specificity in determining iron content in TBI lesions and neurodegenerative patterns (Gozt et al., 2021; Koch et al., 2021; Ravanfar et al., 2021). However, these techniques have limitations, such as the inability to distinguish ferroptosis-specific iron from other forms of iron deposition, underscoring the need for further standardization.

### 4.2 Biofluid biomarkers

Ferroptosis can be diagnosed minimally invasively with biofluid analysis. When compared to controls, AD patients have greater levels of lipid peroxides in their CSF (Mizoi et al., 2014; Bradley-Whitman and Lovell, 2015; Plascencia-Villa and Perry, 2023). After TBI, GPX4 activity is significantly decreased in serum and CSF (Cheng et al., 2023; Fang et al., 2023). Free iron levels in CSF correlate with the severity of SCI (Kwon et al., 2017). These biomarkers are at various stages of validation, with some undergoing Phase II validation and others being verified across multiple centers.

### 4.3 Histopathological techniques

Histopathological techniques offer a direct assessment of ferroptosis in tissue samples. The semi-quantitative identification of iron in post-mortem tissue is frequently accomplished using Prussian blue staining. The ACSL4/GPX4 ratio has been shown to predict ferroptosis in MS lesions, and 4-HNE adducts indicate lipid peroxidation in the AD hippocampus (Butterfield et al., 2010; Luoqian et al., 2022; Jia et al., 2023). The C11-BODIPY test, which uses a fluorescent probe to selectively sensitize lipid peroxidation in ferroptosis, is one of the emerging techniques in this field (Dai et al., 2023).

### 4.4 Challenges and future directions

Ferroptosis diagnosis still faces a number of difficulties despite tremendous advancements. Clinical interpretation is made more difficult by the absence of established reference ranges for biofluid indicators such as lipid peroxides and CSF iron. Furthermore, it is still unclear how these biomarkers change over time in acute vs. chronic damage phases. It is imperative to build multiplex point-of-care platforms that can concurrently evaluate oxidative stress, inflammation, and iron status in order to address these issues. There is potential for these combined techniques to increase the precision and effectiveness of ferroptosis diagnosis in clinical settings.

## 5 Therapeutic strategies in CNS injuries: preclinical evidence and clinical translation

Targeting ferroptosis therapeutically shows promise for treating CNS injuries. Clinical translation is made more difficult by the fact that intervention efficacy differs greatly between experimental models and disease situations. Preclinical information from *in vitro* and *in vivo* studies of acute injuries and neurodegenerative disorders, as well as clinical practice concerns, are reviewed in conjunction with ferroptosis-targeted therapies. Key agents are summarized in Table 2.

**TABLE 2:** Therapeutic agents inhibiting ferroptosis: mechanisms and clinical advances in CNS injuries.

Therapeutic agent	Mechanism	Models	Outcomes	Clinical relevance	References
Baicalein	Anti–inflammatory, antioxidant; inhibits ferroptosis	TBI mouse model	Reduces TBI damage and memory deficits	Potential for CNS injury treatment, limited clinically	Li et al., 2022; Li et al., 2017
Melatonin	Antioxidant; reduces iron and neurodegeneration	Multiple CNS injury models (inferred)	Protects CNS	Promising for treatment, needs clinical validation	Rui et al., 2021
Polydatin	Antioxidant; inhibits ferroptosis–related changes	TBI mouse model	Protects neurons and motor function	Potential for TBI treatment, not in clinical use	Huang et al., 2021
DFO	Chelates iron, antioxidant	TBI, PD, CH, SCI models	Protects in multiple injury models; PD efficacy unclear	For multiple injury treatment research; efficacy to be verified	Grolez et al., 2015; Martin-Bastida et al., 2017
Deferiprone	Chelates iron	PD	Protects neurons	Clinical potential	Dexter et al., 2011; Negida et al., 2024
Fer-1	Inhibits ferritin deposition, protects neurons	PD, TBI, SCI models	Reduces injury, protects neurons	Clinical potential; mainly pre–clinical research	Zhang X. et al., 2024; Miotto et al., 2020
Lip-1	Inhibits ferroptosis, regulates related factors	TBI model	Reduces TBI damage, improves cognition	Neuroprotective; limited clinical application	Cao et al., 2021
SRS16-86	Inhibits ferroptosis	SCI	Alleviates astrogliosis, protects neurons	New treatment perspective; preclinical stage	Zhang et al., 2019
HBED	Binds ferrous ions, reduces iron damage, eases edema	TBI model	Reduces TBI damage, improves motor function	Potential for TBI treatment; few clinical uses	Khalaf et al., 2018

### 5.1 Interventions validated in cellular models

Several therapies have demonstrated proof-of-concept *in vitro* using glial and neuronal cells. Iron chelators such as DFO (100 μM) have been shown to downregulate TFR1 and reduce erastin-induced ferroptosis in SH-SY5Y dopaminergic neurons (Hadzhieva et al., 2013; Kabiraj et al., 2015). Similarly, GPX4 activity is restored by Lip-1 (0.5 μM), saving HT22 hippocampal neurons treated with RSL3 (Friedmann Angeli et al., 2014). These *in vitro* models yield promising results, but they lack the neuroimmune interaction, systemic metabolic effects, and BBB dynamics necessary to fully understand the therapeutic potential of the medications.

### 5.2 Therapeutic efficacy in animal models

#### 5.2.1 Neurodegenerative disorders

Several compounds have demonstrated significant therapeutic potential in animal models of neurodegenerative diseases, such as AD and PD (Carrera and Cacabelos, 2019). Fer-1, administered intraperitoneally at a dose of 2.5 mg/kg, decreased neuronal loss in the substantia nigra pars compacta and improved rotarod test performance in a mouse model of MPTP-induced PD (Wang et al., 2022). Deferiprone (10 mg/kg) dramatically reduced the loss of dopaminergic neurons and striatal dopamine content in the 6-OHDA rat model of PD (Dexter et al., 2011). Lip-1 given intraperitoneally (10 mg/kg) reduced hippocampus iron and restored impairments in the Morris water maze test in an APP/PS1 mouse model of AD (Hambright et al., 2017). Vitamin E/Lip-1 rescues cognitive decline in AD mice (Hambright et al., 2017). Selenium supplementation increases GPX4 activity, reducing ferroptosis in MS models (Van San et al., 2023). Inhibition of ACSL4 such as thiazolidinediones and rosiglitazone facilitates neurological recovery by regulation of ferroptosis after stroke and TBI (Yang et al., 2023).

#### 5.2.2 Acute CNS injuries

Promising outcomes have also been noted in models of acute CNS injuries, including CH and SCI. After CH, DFO was administered to a rat model, which resulted in improved limb symmetry and a statistically significant decrease in edema (Okauchi et al., 2009). Ferroptosis inhibitor SRS16-86 improved the Basso, Beattie, and Bresnahan locomotor scores and increased GPX4 expression in a rat model of contusion SCI (Zhang et al., 2019). Fer-1 improves SCI recovery compared to the free drug (Liu S. et al., 2024). Nanoparticles co-loaded with DFO and antioxidants show synergistic effects in TBI (Huang et al., 2024).

### 5.3 Clinical translation status

There are various obstacles to overcome while moving from preclinical success to clinical application. Among the trials that have been finished is the use of DFO in PD (Phase II), which appeared to reduce nigral iron on MRI but had no effect on the Unified PD Rating Scale (Martin-Bastida et al., 2017). Deferiprone decreased iron in the substantia nigra and raised striatal dopamine levels when given orally at 30 mg/kg in a phase 2 randomized double-blind placebo-controlled clinical trial in PD (Martin-Bastida et al., 2017). The N, N′-Di(2-hydroxybenzyl) ethylenediamine-N, N′-diacetic acid monohydrochloride (HBED), an iron chelator, mitigates iron-related damage by binding ferrous ions, facilitating their transport across BBB, and converting them to ferric ions. With a higher iron affinity and fewer adverse effects than DFO, HBED has demonstrated benefits in TBI models (Khalaf et al., 2018). Moreover, HBED has passed Phase I in clinical trials and is shown to be safe to administer to humans (Grady et al., 1994; Chang et al., 2005). Lip-1 for TBI has finished Phase I safety investigations (Mohammed et al., 2023). A trial (NCT04566991) will evaluate the safety and efficacy of clinical DFO for the treatment of SAH in patients. However, the narrow therapeutic window and BBB penetration are significant translation challenges.

### 5.4 Comparative therapeutic efficacy

The translation of therapeutic agents from animal models to clinical trials faces significant barriers. Although iron chelators have demonstrated neuroprotection in animal models, their use in human trials has been constrained by insufficient CNS biodistribution. Because of pharmacokinetic instability, GPX4 activators remain in the preclinical stage even though they significantly reduce the extent of lesions in TBI mice (Liu J. et al., 2024; Qian et al., 2025). ACSL4 inhibitors have decreased ferroptosis in AD mice, but their poor target selectivity makes them unsuitable for human trials (Jia et al., 2023; Zha et al., 2025).

### 5.5 Future directions

Future studies should concentrate on combinatorial regimens to improve therapy efficacy, such as combining immunomodulators and ferroptosis inhibitors. Novel delivery methods, such as nanoparticles, which boost brain bioavailability in primates, present encouraging paths around BBB restrictions (Pinheiro et al., 2021). Additionally, increasing the success rate of therapeutic interventions will require the use of biomarker-guided therapy, such as QSM-MRI for patient stratification in clinical trials.

## 6 Conclusion and perspectives

Ferroptosis, a form of programmed cell death characterized by iron-dependent LP, has emerged as a critical factor in CNS injuries. While iron is essential for neurological function, excessive iron accumulation can lead to cellular damage and death. Ferroptosis involves various mechanisms, including iron metabolism, amino acid, lipid metabolisms, and immune responses.

However, the current understanding of the mechanisms of ferroptosis remains incomplete, especially within the complex neurological environment. It is yet unclear how ferroptosis and other cell death mechanisms interact, and research is needed to understand their cross-regulatory networks utilizing genetic engineering models like GPX4/ACSL4 double knockout systems. Additionally, ferroptosis activates immune cells, particularly macrophages and microglia, by inducing inflammatory responses, playing a significant role in CNS pathology. Future studies should focus on exploring the specific mechanisms of immunomodulation during ferroptosis, including how ferroptosis activates microglia and macrophages and promotes inflammatory responses. The development of novel treatments for CNS injury may be facilitated by these investigations. Next-generation ferroptosis treatments should either have better BBB permeability or use delivery systems based on nanoparticles for accurate CNS targeting to improve therapeutic approaches. Ferroptosis biomarkers, such as LP products, should also be included in clinical trial designs to precisely evaluate the therapeutic effects of anti-ferroptosis medication. By gaining a deeper insight into ferroptosis and its associated immune mechanisms, we can potentially uncover more effective treatments for CNS injuries and other conditions linked to ferroptosis.
